# Antibiotic Prescription Patterns for Endodontic Procedures in India: A Knowledge, Attitude, and Practices (KAP) Survey

**DOI:** 10.7759/cureus.37804

**Published:** 2023-04-18

**Authors:** Ramya Vengidesh, Sadasiva Kadandale, Anupama Ramachandran, Srividhya Srinivasan, Revathy Parthasarathy, Yashini Thanikachalam, Praveen Kumar

**Affiliations:** 1 Conservative Dentistry and Endodontics, Chettinad Dental College and Research Institute, Chennai, IND; 2 Conservative Dentistry and Endodontics, Sri Venkateswara Dental College and Hospital, Chennai, IND; 3 Conservative Dentistry and Endodontics, Sree Balaji Dental College, Chennai, IND

**Keywords:** systemic antibiotics, empirical prescriptions, kap survey, local antibiotics, aware classification, antimicrobial stewardship, antibiotic over-prescription, antibiotic resistance

## Abstract

Objective

The purpose of this study is to evaluate the antibiotic prescription patterns of endodontists, general dentists, and other dental specialists for endodontic procedures in India by using the knowledge, attitude, and practices (KAP) survey method.

Methods

This cross-sectional study was carried out from February 2022 to May 2022 and involved dentists across India. A self-made questionnaire survey was created to judge the knowledge of various dental practitioners, which includes general dentists, endodontists, other dental specialists, and post-graduates with regard to antibiotic usage guidelines for endodontic purposes. A total of about 310 dental practitioners were surveyed across India. The questionnaire was circulated via social platforms such as WhatsApp, Instagram, and Facebook Messenger.

Statistical analysis

Data for KAP regarding antibiotic prescription patterns among general dentists, endodontists, other dental specialists, and postgraduates were entered into Microsoft Excel and analyzed using IBM SPSS Statistics for Windows, Version 20 (IBM Corp. Released 2011. IBM SPSS Statistics for Windows, Version 20.0. Armonk, NY: IBM Corp.). Descriptive statistics of the study population were examined. The level of statistical significance was determined at p<0.05.

Results

For the percentage of patients who were prescribed systemic antibiotics every day for endodontic reasons, about 38.6% (119) responded 0-10% and 27.3% (84) responded 10-30%. For the order of antibiotics that they prefer from most to least, about 85.4% (263) responded amoxicillin > metronidazole > doxycycline > azithromycin > clindamycin > ciprofloxacin. For the question of whether they use local antibiotics, about 35% answered yes of which 25% were endodontists, 2% were general dentists, 5% were other dental specialists, and 3% were post-graduates. About 77.3% of the total participants were unaware of the antimicrobial stewardship concept and AwaRe classification from WHO. About 53.2% (164) attended CDE programs with regard to antibiotic usage.

Conclusion

It is evident from the results of the present study that there is over-prescription of antibiotics by practitioners especially by general dentists without following proper guidelines for endodontic treatments. More emphasis should be made on the proper prescription pattern of antibiotics, proper understanding of endodontic diagnosis, and the need for antibiotics at the undergraduate level. In addition, proper awareness, as well as proper prescription of antibiotics, should be made for existing dental professionals.

## Introduction

Antibiotics were introduced in the late 1920s. These life-saving medicines should be used appropriately, or they would result in the development of resistance. Unfortunately, antibiotic over-prescription has resulted in the development of newer strains of bacteria that are resistant to these antibiotics. A large number of antibiotics that were effective in the past are not prescribed nowadays because of the development of resistance [1].

Agnihotry et al. (2019) stated that the prescription of systemic antibiotics has become a common part of dental practice and has increased to a greater extent in the last two decades. Dental antibiotic prescription accounts for about 7-10% of the total antibiotic that has been prescribed worldwide for other medical reasons [2]. The inappropriate use of antibiotics produces superbugs that render fatal infections in susceptible individuals. The absence of solid scientific evidence that states the clinical situations, which require the systemic supply of antibiotics, is the major reason for over-prescription [3]. The overuse of antibiotics has become more common during the COVID-19 pandemic. A systematic review concluded that self-medication of antibiotics increased by up to 88% in low- and middle-income countries [4]. In addition, a study from India reported that 216 million additional doses of antibiotics were sold during the pandemic 2020, which would lead to an increase in antibiotic resistance [5].

Clinical situations that require antibiotic therapy include oral infections in medically compromised patients and those with systemic involvement such as malaise, elevated body temperature, trismus, lymphadenopathy, and progressive and persistent infections. Endodontic conditions, such as symptomatic reversible and irreversible pulpitis, pulpal necrosis, acute apical periodontitis, acute apical abscess with no systemic involvement, and chronic apical abscess, do not require antibiotic coverage [6].

The primary cause of pulpal and periapical pathosis is micro-organisms [7]. Systemic antibacterial medication is not required in every endodontically involved tooth. They can be successfully managed by pulp extirpation and proper mechanical and chemical debridement of the canals. The use of antibiotics for immediate pain relief in teeth with acute pulpitis has no proven benefits [8].

Abuse and irrational prescription are the main contributing factors to the emergence of antimicrobial resistance (AMR). Hence, the primary goal of this knowledge, attitude, and practices (KAP) survey is to assess the antibiotic prescription pattern among general dentists, endodontists, other dental specialty persons, and post-graduates in India for endodontic procedures.

The null hypothesis is that there is no difference in the KAP regarding antibiotic prescription patterns among endodontists and other dentists.

The research hypothesis is that there is a difference in the KAP regarding antibiotic prescription patterns among endodontists and other dentists.

## Materials and methods

This cross-sectional study was carried out from February 2022 to May 2022 and involved dentists across India. Ethical approval was obtained from the Institutional Human Ethics Committee registered with the Central Drugs Standard Control Organisation (reg. no.: ECR/212/Inst/TN/2013/RR-19) of Chettinad Academy of Research and Education (proposal No. IHEC-I/0831/22). The purpose of this study is to evaluate dental professionals' KAP about the usage of antibiotics for endodontic reasons. The current study's inclusion criteria include general dentists, endodontists, other dental specialists, and post-graduates of all dental specialties in India. In addition, dental professionals who perform endodontic procedures in their day-to-day practice were only included in the survey. Practitioners who refused to take part in the study and those who do not perform endodontic procedures were excluded.

A self-made questionnaire survey was created to judge the knowledge of various dental practitioners with regard to antibiotic usage guidelines for endodontic purposes. The questionnaire consisted of two parts: Part A contained demographic details and Part B contained the questionnaire proper with 16 questions regarding endodontic practice. They were presented as follows: six multiple-choice questions, one open-ended question, and nine yes/ no questions.

The sample size of the current study was estimated using statistical power analysis G*Power software (version 3.1.9.2) and considering χ² tests, goodness-of-fit tests, contingency tables, and other input parameters:

1. α error as 0.05 at 95% CI

2. β error as 0.20

3. Power of the test (1-β error) as 80%

4. Degrees of freedom as 4

5. Effect size (Cohen’s w statistic) as 0.1880 (determined by Parekh et al. (2020))

The required total sample size for the current study was 338 participants with an output actual power of 80.04%. The questionnaire was circulated based on 338 participants, but only 310 participants responded to the questionnaire, thereby having a non-response rate of 8.87%. The questionnaire was circulated via social platforms like WhatsApp, Instagram, and Facebook Messenger.

## Results

Data regarding KAP regarding antibiotic prescription patterns among general dentists, endodontists, other dental specialists, and postgraduates were entered into Microsoft Excel and analyzed using IBM SPSS Statistics for Windows, Version 20. The descriptive statistics of the study population were examined. The relationship between the designation and years of experience and KAP regarding antibiotic usage was analyzed using the Chi-square test. The level of statistical significance was determined at p<0.05.

In this study, among 308 dentists who answered the questionnaire survey, 32.5% (100) were general dentists, 24.4% were from other specialty PGs, 20.8% were endodontic PGs, 13% were endodontists, and only 9.4% were other dental specialists of which 2% were pedodontists, 2% were prosthodontists, 1.2% were orthodontists, 1.2% were oral surgeons, 1% were oral pathologists, 1% were periodontists, and the remaining 1% were public health dentists. In addition, 77.6% (239) had less than five years of clinical experience, 14.6% had 5-15 years of clinical experience, and only 7.8% had more than 15 years of clinical experience (Table 1).

**Table 1:** Descriptive statistics of the study population

Demographic Characteristics (n = 308)		n	Percent (%)
Gender	Male	105	34.1
	Female	203	65.9
Age group	20-30 yrs	249	80.8
	31-40 yrs	36	11.7
	41-50 yrs	17	5.5
	51-60 yrs	4	1.3
	61-70 yrs	2	0.6
Designation	General dentist	100	32.5
	Endodontist	40	13.0
	PGs in endodontics	64	20.8
	Other dental specialists	29	9.4
	Other PGs	75	24.4
Years of clinical experience	<5 yrs	239	77.6
	5-15 yrs	45	14.6
	>15 yrs	24	7.8

For the total percentage of endodontic cases they handle on average in a day during their clinical practice, about 50.3% (155) responded 0-5, and 35.7% (110) responded 6-10. For the percentage of patients who were prescribed systemic antibiotics every day for endodontic reasons, about 38.6% (119) responded 0-10%, and 27.3% (84) responded 10-30%. For the question conditions for which antibiotics were being prescribed, about 15.9% (49) responded dentoalveolar abscess, replantation after avulsion, and 84.1% (259) responded pain relief, reversible pulpitis, irreversible pulpitis, and endodontic flareups. For the most commonly prescribed antibiotics, about 87%(268) responded amoxicillin, and 11% (34) responded metronidazole. In the clinical situation for which they prescribe antibiotics, about 41.9% (129) responded based on radiological findings like periapical lesions and 25%(77) responded that they would prescribe in the presence of systemic disorders like diabetes mellitus, hypertension, and cardiac conditions. For the order of antibiotics they would prefer from most to least, about 85.4% (263) responded amoxicillin > metronidazole > doxycycline > azithromycin > clindamycin > ciprofloxacin. For the question of whether they use local antibiotics, about 35% answered yes of which 25% were endodontists, 2% were general dentists, 5% were other dental specialists, and 3% were post-graduates.

Regarding the prophylactic prescription of antibiotics prior to apical surgery, 76.6% (236) responded "yes," and 84.7% (261) responded "no." For the question of whether they advise antibiotic culture tests for their patients, 61.4% (189) responded that their patients had self-prescribed antibiotics, and 50% responded that their patients haven’t responded to the prescribed antibiotics. With regard to the use of a combination of antibiotics for synergistic effects, 70.1% (216) responded "yes," 67.5% (208) prescribed drugs based on drug dosage formula, half-life, and weight of the patient, and 71.8% (221) upgraded themselves with the new guidelines and updates regarding antibiotics prescription patterns. About 77.3% of the total participants were unaware of the AMS concept and AwaRe classification from WHO. About 53.2% (164) have attended CDE programs with regard to antibiotic usage.

## Discussion

A recent study by the American Association of Endodontists (AAE) states that general dentists perform the majority of root canal treatments when compared with endodontists. Studies have reported that about 10 million deaths would occur per year globally by 2050, which makes drug resistance a curse of the medical profession unless sustained actions are not taken [9].

Various contributing factors for AMR include inappropriate antibiotic prescription by medical or dental practitioners [2]. Once resistance is formed, reversal of it is impossible, but we shall minimize the development of new resistant strains by proper use of antibiotics [10].

The British Society for Antimicrobial Chemotherapy states that improper antibacterial drug prescription by dental practitioners is a major contributing factor in the development of drug-resistant strains. Factors such as inappropriate dosing, duration, and prophylaxis may contribute to the development of resistant strains. Therefore, it is essential to evaluate the prescription pattern in India to know whether there is a need for continuous education on antibiotic prescription patterns for dentists [11].

AMR has been acknowledged as a global danger to public health by the World Health Assembly's acceptance of the global action plan on AMR in May 2015 and the political statement of the high-level meeting of the general assembly on AMR in September 2017 [12].

This study shows that though a large number of respondents 38.6% (119) found the requirement to prescribe antibiotics was only 0-10% of the endodontic patients who have been examined every day, there were also 27.3% (84) who prescribed antibiotics in the range of 10-30% and 19.5% (60) who prescribed antibiotics between 30% and 50%, which may contribute to over-prescription of antibiotic to some extent. In addition, the majority of general dentists prescribe antibiotics for 10-30% of endodontic cases they handle in their day-to-day practice. This may be due to anxiety about pain development, preventing endodontic flareups, improving patient comfort, and the lack of awareness of proper antibiotic prescription patterns.

According to a systematic review by James et al., antibiotics were not essential for irreversible pulpitis and pain relief [13,14]. In line with this, the current study found antibiotic misuse to some extent by prescribing them for pain relief, reversible pulpitis, irreversible pulpitis, and endodontic flareups (84.1%). The guidelines of the American Dental Association state that antibiotic usage for dental conditions must be limited to situations only when there is systemic involvement and immediate, definitive as well as conservative dental treatment should be performed in all cases [15]. About 35% of participants responded that they would use local antibiotics, but the position statement on the use of antibiotics in endodontics which was given by the European Society of Endodontology (ESE) states that there is no scientific evidence that supports the use of topical antibiotics for root canal disinfection [6].

About 87% of participants chose Amoxicillin as the first line of drug, followed by metronidazole (11%). This is consistent with the study done by Maslamani et al. [16]. Amoxicillin, a synthetic improvement of the penicillin molecule, is readily absorbed when taken with food and is resistant to damage by stomach acid. However, amoxicillin has a broad spectrum of activity compared with penicillin [17]. Metronidazole has been suggested to be taken with amoxicillin due to its very good activity against anaerobes [18].

The 2017 AAE guidelines regarding antibiotic prophylaxis given recommend prescribing antibiotics for diabetic patients with poor glycemic control [19]. The ESE position statement that was given on the use of antibiotics in endodontics recommends antibiotic prophylaxis in medically compromised patients with acute apical abscess and those cases with systemic involvement, progressive infections, replantation of avulsed teeth (permanent teeth), soft tissue trauma [6]. The American Heart Association (AHA) 2007 guidelines, which were revised from earlier ones for cardiac conditions, state that antibiotic prophylaxis is essential only for patients with high-risk infective endocarditis, and this prophylaxis is essential for dental procedures that involve the handling of gingival tissues, oral mucosa, or periapical part of teeth [20]. In addition, the AHA's recent 2021 scientific update recommends antibiotic prophylaxis in cases with prosthetic cardiac valve/material, congenital heart disease, and cardiac transplant recipients who develop cardiac valvulopathy.

Studies conducted by Amisha et al. 2020 and Singh et al. found that <40% of dentists have advised antibiotic culture test, and in this study, only 15.3% of dentists have advised it [21,22]. Furthermore, about 61.4% of patients have self-prescribed antibiotics, and 50% of patients haven’t responded to the antibiotics which is almost similar to the results of the study done by Amisha et al. This provides us the hint that we should focus on antibiotic culture tests.

A combination of antibiotics is prescribed by more than 70% of dentists. Amoxicillin + clavulanic acid is one such combination that may be used for serious oral infections and in cases where resistant species might be suspected, which is refractory to normal endodontic procedures [23].

The antimicrobial stewardship (AMS) concept was introduced by WHO to take care of the health and well-being of the people and provide guidelines for health systems both nationally and globally. The three pillars that strengthen the health systems are AMS, medicine and patient safety, and infection prevention and control. AMR shall be controlled with the help of AMS, antibiotic surveillance, the AWaRe classification, and the essential medicine list (EMS) from WHO [12]. In AWaRe classification, the antibiotics are grouped into three different categories: access, watch, and reserve group. Access group antibiotics have a wide range of activity against commonly encountered pathogens. In addition, they have a lower tendency for resistance than other groups. Watch group antibiotics have a higher tendency of resistance, and they should be the key targets of local and national stewardship programs. Reserve group antibiotics should be used for the treatment of suspected or confirmed infections because of multidrug-resistant organisms, and they are the last resort option and should also be the key target of stewardship programs [12].

In this study, about 77% of dentists were unaware of the AMS concept and AWaRe classification from WHO, and this clearly states the reason for the overuse or misuse of drugs. In addition, 53.2% of dentists have not regularly attended CDE programs with regard to antibiotic resistance.

The endodontic flareups shall be prevented by following the proper working length of the tooth being prepared, thereby preventing apical extrusion of debris. NiTi rotary instruments showed less debris extrusion when compared with the step-back technique using manual instruments [24]. Furthermore, rotary instruments produced less extrusion of debris when compared with reciprocating instruments [25]. Anti-microbial sensitivity testing should be done on susceptible patients using recent sensitivity testing methods such as phenotypic and genotypic rapid antimicrobial sensitivity testing and image-based antimicrobial sensitivity testing [26].

## Conclusions

It is evident from the results of the present study that there is an over-prescription of antibiotics by practitioners, especially by general dentists, without following proper guidelines for endodontic treatments. More emphasis should be made on the proper prescription pattern of antibiotics at the undergraduate level. In addition, dental practitioners must be updated on recent guidelines for antibiotic prescription, the WHO’s EML, and the AWaRe classification with the help of CDE programs and by following proper endodontic diagnosis and treatment protocols to prevent endodontic flareups, thereby reducing the need for antibiotics. Patients should be warned about the adverse effects of self-prescribing antibiotics.

## Appendices

1. How many patients on average do you handle in your day-to-day practice?

0-5

6-10

11-15

16-20

2. Percentage of patients who were prescribed systemic antibiotics every day.

0-10%

10-30%

30-50%

50-80%

3. What are the following conditions do you prefer to prescribe antibiotics?

Pain relief

Reversible pulpitis

Irreversible pulpitis

Endodontic flareups

Dentoalveolar abscess

Replantation after avulsion

4. Which of the following antibiotics is the most commonly prescribed one?

Amoxicillin

Metronidazole

Doxycycline

Clindamycin

Azithromycin

Ciprofloxacin

5. Choose one among the following factors based on which you prescribe antibiotics.

Systemic illnesses like diabetes mellitus, hypertension, and cardiac disease

To prevent flare-ups after root canal treatment

Size of the swelling

Based on radiological findings like periapical lesions

6. Choose the order of antibiotics that you prefer from most to least.

Amoxicillin > metronidazole > doxycycline > azithromycin > clindamycin > ciprofloxacin

Metronidazole > amoxicillin > doxycycline > azithromycin > clindamycin > ciprofloxacin

Doxycycline > amoxicillin > metronidazole > azithromycin > clindamycin > ciprofloxacin

Azithromycin > amoxicillin > metronidazole > doxycycline > clindamycin > ciprofloxacin

Clindamycin > amoxicillin > metronidazole > doxycycline > azithromycin > ciprofloxacin

Ciprofloxacin > amoxicillin > metronidazole > doxycycline > azithromycin > clindamycin any order of your preference

7. Do you prescribe antibiotics prior to surgery prophylactically?

Yes

No

8. Do you advise antibiotic culture tests for your patients?

Yes

No

9. Have your patients ever self-prescribed antibiotics?

Yes

No

10. Do any of your patients haven’t responded to the antibiotics that you’ve prescribed?

Yes

No

11. If yes, what is the reason for not responding to antibiotics?

Not sensitive

May require a better anti-inflammatory to reduce clinical symptoms

The patient might have developed tolerance toward the drug

Systemic factors might have resulted in a need for increased dosage

12. Any combinations of antibiotics/local antibiotics you use for synergistic effects?

Yes

No

13. Do you prescribe the drugs based on the drug dosage formula, half-life, and weight of the patient?

Yes

No

14. Do you upgrade yourselves with the new guidelines or updates regarding antibiotic usage?

Yes

No

15. Do you have any idea about the antimicrobial stewardship concept and "AWaRe" classification from WHO?

Yes

No

16. Have you attended or listened to any CDE programs with regard to antibiotic resistance?

Yes

No
